# Habitat-Specific Density and Diet of Rapidly Expanding Invasive Red Lionfish, *Pterois volitans*, Populations in the Northern Gulf of Mexico

**DOI:** 10.1371/journal.pone.0105852

**Published:** 2014-08-29

**Authors:** Kristen A. Dahl, William F. Patterson

**Affiliations:** University of South Alabama, Department of Marine Sciences, Mobile, Alabama, United States of America; North Carolina State University, United States of America

## Abstract

Invasive Indo-Pacific red lionfish, *Pterois volitans*, were first reported in the northern Gulf of Mexico (nGOM) in summer 2010. To examine potential impacts on native reef fish communities, lionfish density and size distributions were estimated from fall 2010 to fall 2013 with a remotely operated vehicle at natural (n = 16) and artificial (n = 22) reef sites. Lionfish (n = 934) also were sampled via spearfishing to examine effects of habitat type, season, and fish size on their diet and trophic ecology. There was an exponential increase in lionfish density at both natural and artificial reefs over the study period. By fall 2013, mean lionfish density at artificial reefs (14.7 fish 100 m^−2^) was two orders of magnitude higher than at natural reefs (0.49 fish 100 m^−2^), and already was among the highest reported in the western Atlantic. Lionfish diet was significantly different among habitats, seasons, and size classes, with smaller (<250 mm total length) fish consuming more benthic invertebrates and the diet of lionfish sampled from artificial reefs being composed predominantly of non-reef associated prey. The ontogenetic shift in lionfish feeding ecology was consistent with δ^15^N values of white muscle tissue that were positively related to total length. Overall, diet results indicate lionfish are generalist mesopredators in the nGOM that become more piscivorous at larger size. However, lionfish diet was much more varied at artificial reef sites where they clearly were foraging on open substrates away from reef structure. These results have important implications for tracking the lionfish invasion in the nGOM, as well as estimating potential direct and indirect impacts on native reef fish communities in this region.

## Introduction

Introduction of exotic species to marine ecosystems has been increasing in frequency and severity around the globe, which has also lead to an increase in species invasions [1], [2]. Invasive species can significantly transform recipient communities where they reduce biodiversity, displace native species, alter community structure, or introduce pathogens [3], [4], [5], [6]. Anthropogenic activities, namely trade, commerce, and aquaculture, are escalating the rate of species introductions, furthering the need for research on the prevention and mitigation of potential ecological and socioeconomic impacts from invasions [7], [8]. The severity of ecological impacts depends on the life history and trophic dynamics of the invader, as well as the ecology of the invaded community [9], [10]. Predator-prey interactions are known to shape community assemblages in both terrestrial and marine systems [11], [12], thus predator invasions are expected to have the most damaging impact on native ecosystems [13], [14], [15].

Predator invasions in marine ecosystems are atypical, yet the invasion of Indo-Pacific lionfishes (*Pterois volitans/miles*) complex in the western Atlantic Ocean has been so extensive and rapid that lionfish are considered the most successful marine fish invaders to date [16], [17], [18]. Lionfish have established an invaded area over 7 million km^2^ that includes the US Southeast Atlantic coast, the Caribbean Sea and portions of the Gulf of Mexico (GOM) [19], [20], [21]. The GOM is the most recently invaded of these basins, where lionfish were not reported until 2009 off the northern Yucatan peninsula, Mexico [22]. Red lionfish, *Pterois volitans* (hereafter lionfish), were first reported from northern GOM (nGOM) in summer 2010 and have since been observed in the western GOM as well [20], [23], [24].

Several life history and behavioral traits of lionfish are thought to facilitate their continued spread and population growth. For example, lionfish are voracious, novel predators that consume a wide variety of naïve prey in the western Atlantic, but experience little to no predation themselves, in part due to the presence of large, venomous dorsal, pelvic and anal spines [25], [26], [27]. Lionfish can reach sexual maturity within one year [25] and have a high reproductive output [28], [29]. Therefore, lionfish populations in their invaded range have the potential to reach far greater densities than those reported in the Indo-Pacific [30], [31], [32], [33]. Among invaded western Atlantic reef communities, lionfish have had damaging effects on native fishes due to the direct consumption of a broad array of native fishes, including some economically important reef fishes [34], [35]. However, the majority of lionfish impact assessments to date have come from south Atlantic hardbottom [36] or coral reef habitats [37], [27], that are quite different ecologically from nGOM reef habitats. This is especially true for artificial reefs which have been deployed throughout the nGOM and whose reef fish communities have much lower densities of small demersal fishes (e.g., damselfishes, blennies, gobies, and wrasses) [38], [39], that have been shown to be the preferred prey among lionfish sampled at natural reefs throughout the Caribbean [16].

Local research is essential to estimate the direct impacts of invasive lionfish on native fishes, thus an understanding of lionfish diet on both natural and artificial reefs is necessary to predict their impacts in the nGOM. The first objective of this study was to document the progression of the lionfish invasion in an area of the nGOM by monitoring lionfish densities among natural and artificial reef habitats. We also characterized lionfish feeding ecology in the region to recognize potential direct and indirect impacts of lionfish on native reef fish communities of the northern GOM. Stomach content analysis was employed to test for seasonal and ontogenetic effects on lionfish diet between natural and artificial reefs. Traditional diet analysis relies on recently ingested prey, thus was complimented by stable isotope analysis of lionfish white muscle tissue revealing isotopic dietary signals integrated over the previous weeks to months. Results discussed below have implications for predicting both direct and indirect effects of invasive lionfish on natural and artificial reefs in the nGOM.

## Methods

### Ethics Statement

All fish sampled in this study were handled in strict accordance with the laws of the state of Alabama and under the IACUC protocols (Permit Number: 276018) approved by the University of South Alabama. Locations used for both density surveys and lionfish sample collection did not require the use of any specific permissions. No endangered or protected species were involved in this study.

### Lionfish Density Estimates

**Figure 1:**
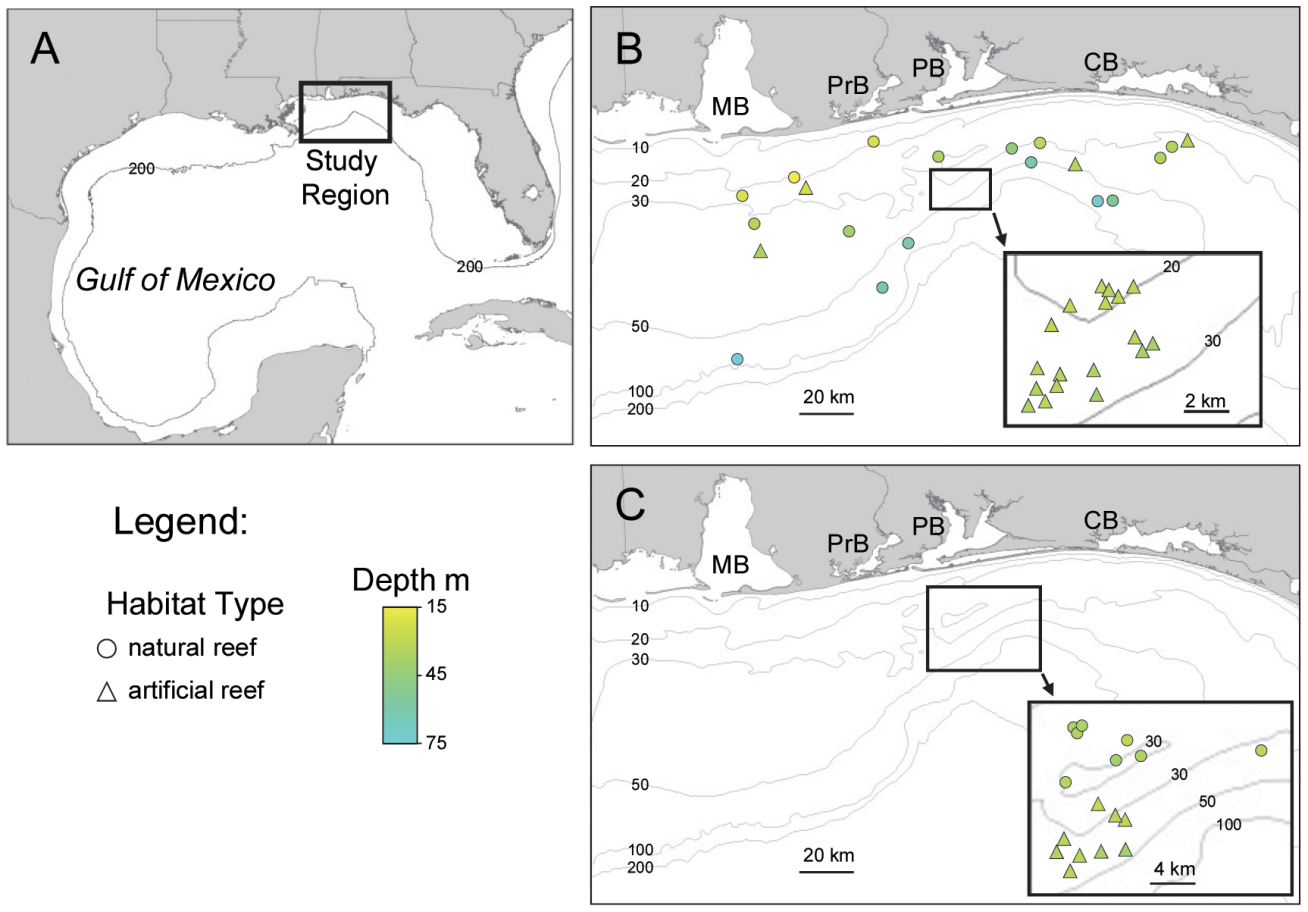
Location of study sites for lionfish density and diet sampling. A) Map of the Gulf of Mexico with study region indicated. B) Natural (circles) and artificial (triangles) reef sites sampled with a remotely operated vehicle in fall 2010–2013 to estimate invasive lionfish density; water bodies: MB  =  Mobile Bay, PrB  =  Perdido Bay, PB  =  Pensacola Bay, and CB  =  Choctawhatchee Bay. C) Natural (circles) and artificial (triangles) reef sites sampled by divers who speared lionfish for stomach content and muscle stable isotope analysis.

Northern GOM natural (n = 16) and artificial (n = 22) reefs were surveyed with a micro remotely operated vehicle (ROV) each fall (October to December) from 2009 through 2013 to examine changes in lionfish density and size distribution over time (Fig. 1B). Reefs were randomly selected from a larger sample frame of regional reefs [38], [39], and ranged in depth between 17 and 73 m. Sampling was conducted with a VideoRay Pro4 ROV (dimensions: 36 cm long, 28 cm tall, 22 cm wide; mass = 4.8 kg). The ROV has a depth rating of 170 m, a 570-line color camera with wide angle (116°) lens, and was equipped with a red laser scaler to estimate fish size. The laser scaler consisted of two 5-mw @ 635 nm (red) class IIIa lasers mounted in a fixed position 75 mm apart. The ROV was tethered to the surface where it was controlled by a pilot via an integrated control box that contains a 38-cm video monitor to observe and capture digital video captured by the ROV's camera during sampling.

Video sampling was conducted at study reefs with either a point-count or transect method, depending on habitat type and dimensions. The point-count method, which is described by Patterson et al. [40], was used to sample a 15-m cylinder around isolated reef habitat, such as single artificial reef modules. In that method, the ROV was positioned 1 m above the seafloor and approximately 5 m away from a given reef. The ROV was slowly pivoted 360° and then moved to the opposite side of the reef. Once there, it was again positioned 1 m above the seafloor and approximately 5 m away from the reef and pivoted 360°. The ROV then was flown to 1 m directly above the reef and pivoted 360° to video fishes in the water column above the reef. Next, the ROV was flown to 10 m above the reef and pivoted 360°. Once all sample segments were completed, the ROV was flown back down to the reef to observe fishes located on the reef's surface or inside the reef structure.

A transect sampling method was utilized for reef habitat that was more broadly distributed, such as was characteristic of natural reef habitat examined in this study. In this method, a 5-m wide transect was video sampled as the ROV moved forward at a rate of approximately 0.5 m s^−1^ along a 25-m long transect. The width of the transect was controlled by flying the ROV with a camera angle of 45° approximately 1 m above the seabed given the 116° viewing angle of the camera [39]. Four orthogonal transects were flown over natural reef habitats, thus a total area of approximately 500 m^2^ of reef habitat was surveyed. The distance covered on a given transect was controlled by flying the ROV with a fixed scope of tether away from a 5-kg clump weight attached in-line to the tether. Transect distance was confirmed with a Tritech MicronNav ultrashort baseline acoustic positioning system deployed with the ROV.

Analysis of video samples was performed with a Sony DVCAM DSR-11 digital VCR and a Sony LMD-170 high resolution LCD monitor. When the point-count method was employed, lionfish counts were summed among all sampling segments and then divided by the sample area (176.7 m^2^) to estimate fish density. Lionfish density for transect samples was computed by summing counts and then dividing by the total area estimated to have been sampled among transects. Total length (TL) was estimated for lionfish struck by the red dots of the laser scaler by first multiplying the length of a fish measured in a video frame by the known distance between lasers (75 mm), and then dividing that product by the distance measured between lasers in the frame. Patterson et al. [40] estimated a mean negative bias of 3% (SD = 0.6) resulted from this method, thus estimated lionfish TL was bias-corrected based on a random probability draw and normally distributed bias with mean equal to 3% and a standard deviation of 0.6%.

The difference in lionfish density between natural versus artificial habitats and among years was tested with a two-factor analysis of variance (ANOVA) model, with Tukey's multiple comparison procedure computed to test all pairwise comparisons. Too few TL estimates were available to test the habitat effect, thus TL was pooled between artificial and natural reefs and the effect of year was tested with a single-factor ANOVA model, with Tukey's multiple comparison procedure computed to test all pairwise comparisons. A priori, α was set to 0.05 for all statistical tests.

### Sampling Lionfish Tissues

Lionfish were sampled by divers with spears to examine lionfish trophic ecology at nGOM reefs. Dive trips were made seasonally from April 2013 through March 2014 to both natural and artificial reefs, with sampling reefs ranging in depth from 24 to 35 m (Fig. 1C). Spearing of lionfish was localized immediately posterior to the head which severed their spinal column. Fish were dead upon arrival to the surface where carcasses were placed in mesh bags in an ice-slurry. Once on land, fish were ranked by size and systematic random sampling was employed to sample every n^th^ fish such that approximately 100 fish were sampled per habitat type per season. Lionfish samples were weighed to the nearest 0.1 g and measured to the nearest mm TL. Approximately 30 g of white muscle tissue was dissected from each fish above its pectoral fin. Muscle tissue was placed in plastic bags and frozen at −80°C. Stomachs were dissected after inspecting gills for regurgitated prey and their contents placed in plastic bags, fixed in 100% ethanol.

A non-linear regression (mass  =  aTL^b^) was fit to lionfish mass and TL data. The fitted equation was then employed to predict mass of lionfish scaled with the red laser scaler in ROV video samples. The difference in mean predicted mass among years was tested with ANOVA. Tukey's multiple comparison procedure also was computed to test for differences in predicted mass between each pair of years.

### Diet Analysis

Prey items in stomach samples were sorted to the lowest taxonomic level possible, counted, and then dried at 60°C for at least 48 h to obtain dry mass. Prey taxa were grouped into seven prey categories: shrimps, crabs, other benthic invertebrates, pelagic invertebrates, reef fishes, non-reef benthic fishes, and pelagic fishes. Percent mass and percent number by prey category were computed for each sample that had prey items present. Percent frequency of occurrence (%F) was calculated among fish captured in a given season and from a given habitat type as the number of stomachs containing a particular prey category divided by the number of stomachs with prey present [41]. The index of relative importance (IRI) was then computed as IRI  =  (%M + %N) x %F [42], and %IRI was calculated by dividing the IRI value for each prey category by the sum of the IRI values among all prey categories and multiplying by 100.

The effects of habitat type (natural versus artificial reef), season, and size class (small: <200 mm, medium: 200–250 mm, and large: >250 mm TL) on lionfish diet by %M and %N were tested with three-factor permutational multivariate analysis of variance (PERMANOVA) models computed with the Primer statistical package (ver. 6; [43]). Data were square-root transformed and a dummy variable (value = 1) was added to each sample prior to computing the Bray-Curtis similarity measure among all pairs of samples. Then, PERMANOVA models were computed with 10,000 permutations to test if the pattern observed in Bray-Curtis similarity between habitats, among seasons, or among fish size classes was significantly different from random. All significant effects or interactions observed in PERMANOVA results were further examined via pair-wise PERMANOVA tests.

### Muscle Stable Isotope Analysis

Stable isotope analysis was conducted for lionfish muscle samples collected in spring 2013 and winter 2014. Samples from 8 fish from each habitat in each of these seasons were selected with systematic random sampling for analysis. Muscle tissue samples were dried in an oven at 60°C for at least 48 h, and then ground in a tissue grinder prior to being pulverized with a glass mortar and pestle. Mortars and pestles were rinsed with deionized water and air-dried between samples, while the tissue grinder was wiped free of dried tissue remnants with a lint-free laboratory tissue. Muscle samples were analyzed for δ^13^C and δ^15^N with a Thermo-Finnigan MAT Delta+ Advantage stable isotope ratio-mass spectrometer (SIR-MS) equipped with an elemental analyzer at the Marine Science Institute of the University of California Santa Barbara. Values of δ^13^C are reported relative to the international standard Vienna Peedee Belemnite, and δ^15^N values are reported relative to atmospheric nitrogen, which is isotopically homogenous. Isotope ratios for both C and N are reported in the standard delta notation: δX = [(R_sample_/R_standard_)−1]×1000, where X = ^13^C or ^15^N and R = ^13^C:^12^C or ^15^N:^14^N. Check standards run periodically during the analysis included US Geological Survey standard reference materials 40 and 41 (glutamic acid).

Values of δ^13^C were corrected for %lipid with the regression equation reported by Post et al. [44], for aquatic animals: Δδ^13^C = −3.32+(0.99×C:N), where Δδ^13^C is the correction applied to δ^13^C to account for %lipid and C:N is a proxy for %lipid. Correlation analyses were computed to test the relationship between fish TL and δ^15^N, and between TL and δ^13^C. Differences in TL, δ^13^C, and δ^15^N were tested between natural and artificial reefs and between spring 2013 and winter 2014 were tested with two-factor ANOVA models. In the case of a significant interaction term, all pairwise multiple comparisons were computed with Holm-Sidak tests.

## Results

### Lionfish Density and Size

**Figure 2:**
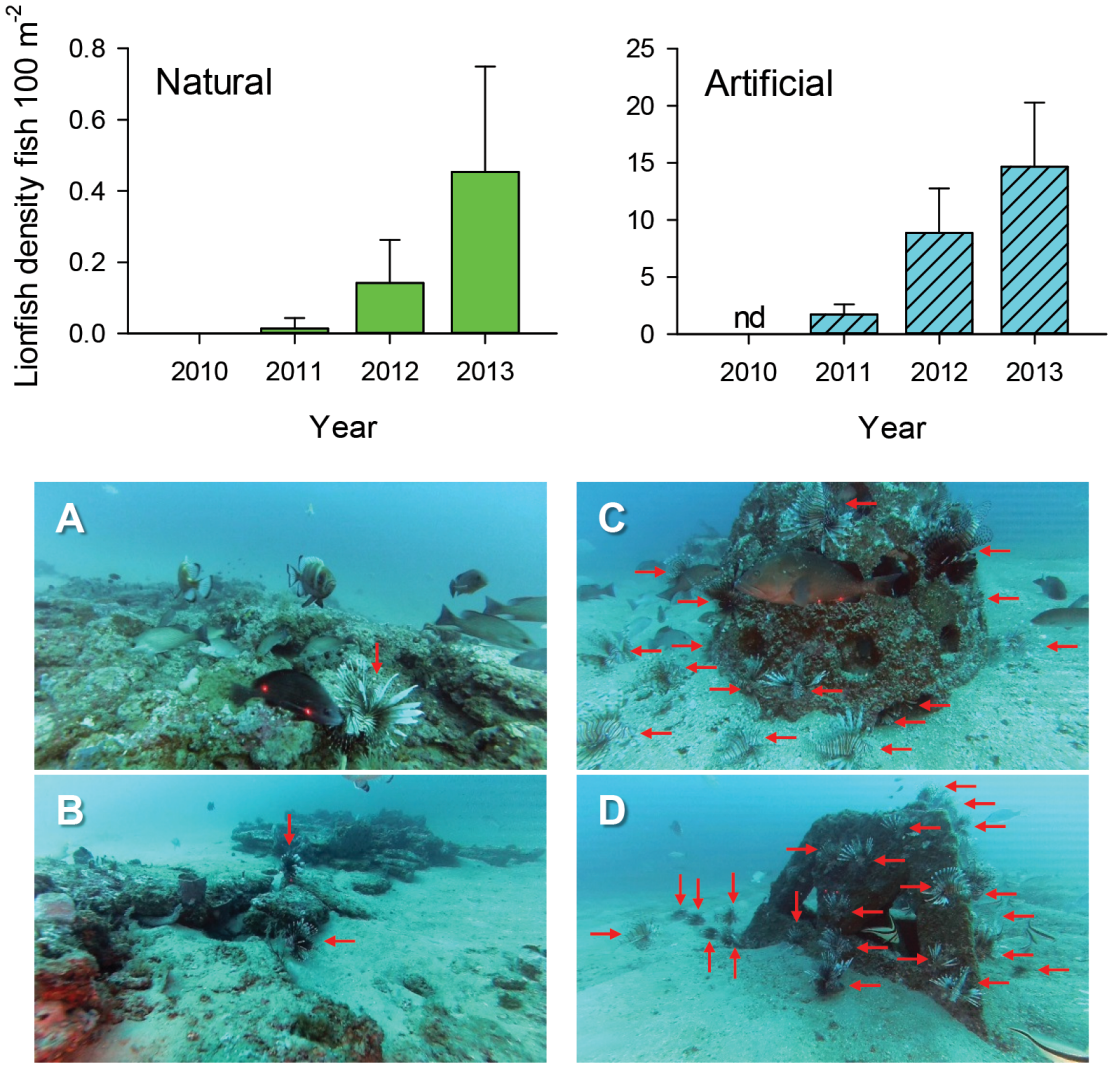
Mean densities of lionfish 100 m^−2^ on natural and artificial reefs. Mean (95% CI) density of lionfish in fall 2010−2013 estimated with micro remotely operated vehicle-based video sampling at northern Gulf of Mexico natural (A,B) and artificial reef sites (C,D); red arrows in reef images indicate lionfish. No video sampling occurred at artificial reef sites in fall 2010.

Lionfish density increased rapidly from fall 2010, when no fish were observed at study reefs (no ROV surveys conducted at artificial reefs in fall 2010), through fall 2013, when mean density was 0.49 fish•100 m^−2^ on natural reefs and 14.7 fish•100 m^−2^ on artificial reefs (Fig. 2). Among all samples, lionfish density ranged from 0 to 1.8 fish•100 m^−2^ on natural reefs and from 0 to 38.5 fish•100 m^−2^ on artificial reefs. Habitat-specific lionfish density estimates violated parametric assumptions, and no transformation was successful in meeting the assumption of normality. ANOVA is robust to violations of normality [45], thus the two-factor model testing the effect of habitat and year on lionfish density was computed with ln-transformed data. Both habitat (ANOVA, F_1;112_ = 44.60, p<0.001) and year (ANOVA, F_2;112_ = 9.56, p<0.001) were significant in the model, but their interaction was significant as well (ANOVA, F_2;112_ = 8.35, p<0.001). The significant interaction was due to more rapid population growth at artificial reefs for which lionfish densities were two orders of magnitude higher than on natural reefs in fall 2013 (Fig. 2).

**Figure 3:**
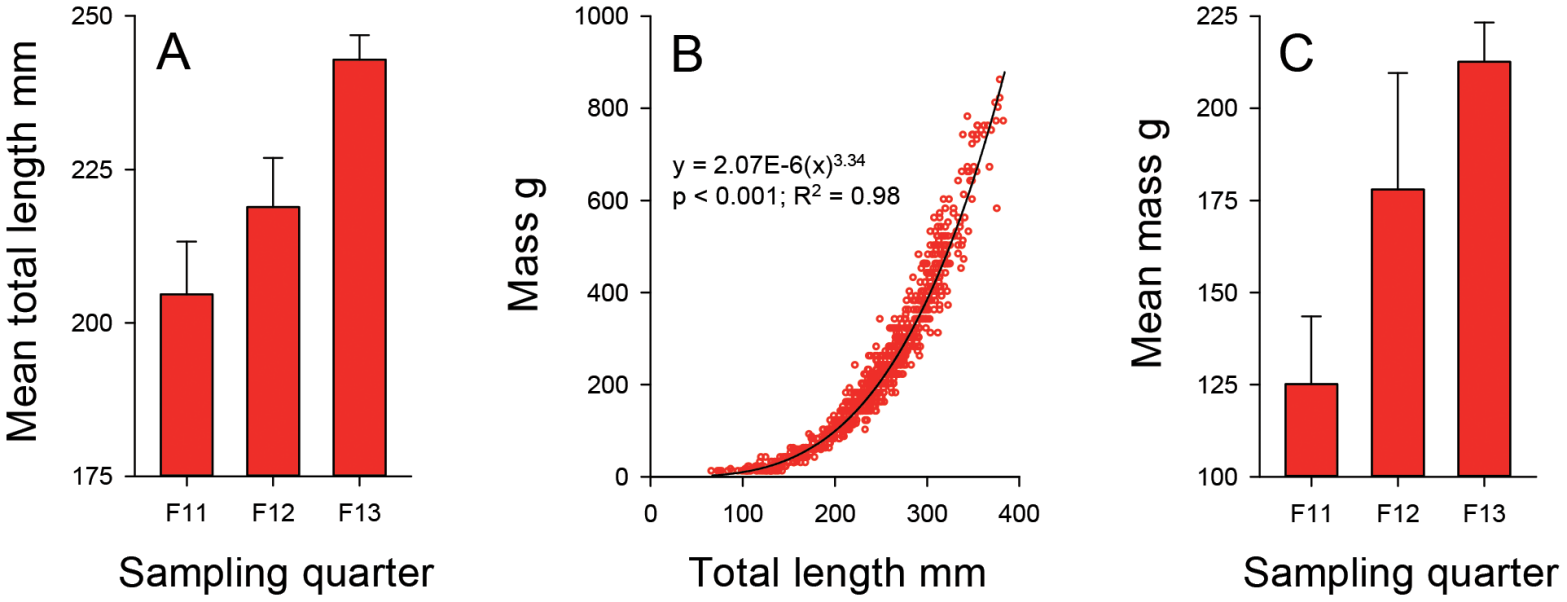
Length to weight relationship of speared lionfish predicts lionfish mass from ROV length observations. A) Mean (95% CI) total length of red lionfish (n = 190) observed in remotely operated vehicle video (ROV) samples at northern Gulf of Mexico reef sites and measured in video images with a red laser scale attached to the ROV; F = fall, 11 = 2011, 12 = 2012, and 13 = 2013. B) Non-linear regression computed to predict red lionfish mass from total length from fish (n = 934) captured by spearfishing. C) Mean (95% CI) predicted mass of lionfish (n = 190) observed in ROV video samples and measured with a red laser scale.

Mean ±95% confidence intervals of lionfish TL estimated with the ROV's laser scale in video samples increased from 204.7±16.9 mm in fall 2011 to 242.9±7.8 in fall 2013 among all study reefs (Fig. 3A). Data were ln-transformed prior to computing the ANOVA testing if fish size was significantly different among years. The model was significant (ANOVA, F_2;187_ = 9.11, p<0.001), and pairwise comparisons were significant between 2011 and 2013 (Tukey's, p<0.001) and 2012 and 2013 (Tukey's, p = 0.007), but not between 2011 and 2012 (Tukey's, p = 0.438). The non-linear regression relating mass to TL for fish (n = 934) sampled by divers with spears was statistically significant (p<0.001) with an adjusted R^2^ of 0.98 (Fig. 3B). Given that fish mass increased faster than TL, the percent increase in predicted mass (69.8%) of fish measured with the ROV's laser scale was greater than the percent increase in TL (18.7%) (Fig. 3C). Predicted lionfish mass was significantly different among years (ANOVA, F_2;187_ = 3.42, p = 0.035), but pairwise comparisons revealed predicted mass was only significantly different between years 2011 and 2013 (Tukey's, p = 0.033) (Fig. 3C).

### Lionfish Diet Analysis

**Figure 4:**
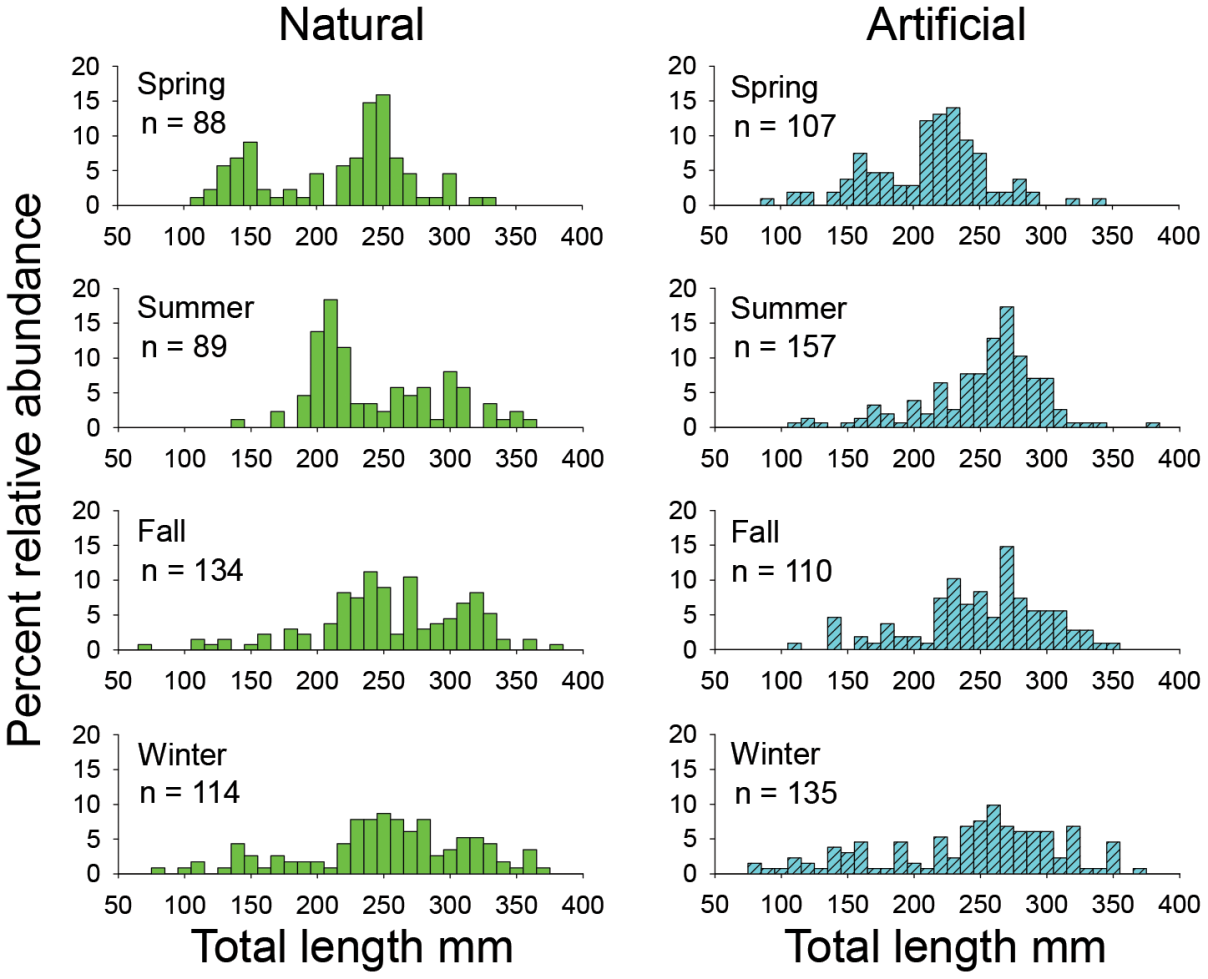
Habitat-specific total length distributions of lionfish samples by season. Season-specific total length distributions of lionfish sampled by spear at northern Gulf of Mexico natural and artificial reef sites from April 2013 through March 2014.

There were 934 lionfish sampled by divers with spears from natural and artificial reefs. Among the 8 habitat-season combinations, sample size ranged from 88 fish at natural reefs in spring 2013 to 157 fish at artificial reefs in summer 2013, with a mean sample size of 117 fish among the combinations. Total length ranged from 67 to 377 mm, with distinct modes in size distributions indicating multiple year classes were likely present among samples (Fig. 4). Among natural reef samples, 85% (361 of 426) had prey present in their stomachs, as did 81% (409 of 508) of artificial reef samples. Among all samples, 43% of lionfish prey by mass was unidentifiable. Identifiable prey consisted of 77 taxa, 39% of which were identified to species (Table 1). Newly reported prey taxa for the northern GOM included two fish families (Family: Plueronectidae, right eye flounders and Family: Paralichthyidae, large tooth flounders) and notable invertebrate taxa, for example, squid (*Loligo* sp.), slipper lobster (Family: Scyllaridae) and Florida stone crab (*Menippe mercenaria*). By mass, the diet of lionfish collected at natural reefs predominantly consisted of reef-associated prey (98.2%), most which (89.5%) consisted of small (<5 cm) demersal reef fishes, such as damselfishes, twospotted cardinalfish (*Apogon pseudomaculatus*), blennies, and wrasses. In contrast, reef-associated prey constituted only 24.4% of lionfish diet at artificial reef sites, which principally consisted of juvenile vermilion snapper (*Rhomboplites aurorubens*), and bank seabass, (*Centropristis ocyurus*). Non-reef benthic fishes, such as lizardfishes, flounders, and searobins, constituted the highest percentage (42.6%) of lionfish diet at artificial reefs, but pelagic jacks and scads (16.3%) and benthic invertebrates (12.3%) were also well-represented.

**Table 1:** Prey taxa observed in red lionfish stomachs sampled in the northern Gulf of Mexico.

Taxon	Common name	Habitat	NR %mass	AR %mass
Crabs				
*Anasimus latus*	silt spider crab		0.00	0.02
Brachyura	crabs		0.07	0.45
Calappidae	box crabs		0.00	0.37
*Calappa sulcata*	yellow box crab	Reef	0.00	0.38
Majidae	spider crabs		0.05	0.07
*Menippe mercenaria*	florida stone crab	Reef	0.14	0.06
*Pagurus* sp.	hermit crab		0.00	0.00
Parthenopidae	elbow crab		0.00	0.01
*Porcellana sigsbeiana*	striped porcelain crab	Reef	0.01	0.00
Portunidae	swimming crab		0.05	0.22
*Portunus sayi*	sargassum swimming crab	Non-reef	0.09	0.75
*Portunus spinicarpus*	longspine swimming crab	Non-reef	0.70	0.19
*Stenorhynchus seticornis*	yellowline arrow crab	Reef	0.00	0.24
Xanthidae	mud crabs	Non-reef	0.08	0.43
Total Crabs			1.19	3.18
Shrimps				
Decapod shrimp	shrimps		1.39	2.75
Alpheidae	snapping shrimps	Reef	0.03	0.04
Caridea	snapping shrimps	Non-reef	0.12	0.31
Penaeidae	penaeid shrimps	Non-reef	2.28	4.12
*Litopenaeus setiferus*	white shrimp	Non-reef	0.27	0.55
*Farfantepenaeus duorarum*	pink shrimp	Non-reef	0.09	0.18
*Trachypenaeus similis*	roughneck shrimp	Non-reef	0.03	0.00
Stenopodidae	cleaner shrimps	Reef	0.00	0.02
Total Shrimps			4.21	7.97
Other Benthic Invertebrates				
Benthic Gastropod	sea snails		0.00	0.16
*Eumunida* sp.	squat lobster	Non-reef	0.51	0.14
Scyllaridae	slipper lobsters	Reef	0.00	0.13
Hippoidea	mole crabs	Non-reef	0.00	0.11
Axiidae	thalassinidean shrimp		0.02	0.11
Decapoda			0.09	0.10
Cumacea	hooded shrimps	Non-reef	0.03	0.00
Squillidae	mantis shrimps	Non-reef	0.21	0.39
*Squilla empusa*	mantis shrimp	Non-reef	0.37	0.00
Octopoda	octopus	Reef	0.01	0.00
Mollusca			0.01	0.00
Pleocyemata			0.07	0.00
Total Other Benthic Invertebrates		1.31	1.14	
Pelagic Invertebrates				
Achelata phyllosoma	larval lobster	Non-reef	0.00	0.01
larval shrimp			0.00	0.01
Euphausiacea	euphausid	Non-reef	0.00	0.02
Gammaridae	amphipod		0.00	0.04
*Loligo* sp.	squid	Non-reef	0.00	4.51
Lophogastrida	pelagic shrimp	Non-reef	0.00	0.07
Mysida	mysid shrimp	Non-reef	0.08	0.02
Total Pelagic Invertebrates		0.08	4.67	
Reef Fishes				
*Apogon* sp.	cardinalfishes	Reef	0.01	0.00
*Apogon pseudomaculatus*	twospotted cardinalfish	Reef	10.35	0.28
Blenniidae	blennies	Reef	7.35	0.17
Gobiidae	gobies	Reef	4.08	0.55
Labridae	wrasses	Reef	1.19	0.06
*Halichoeres bathyphilus*	greenband wrasse	Reef	11.11	0.00
*Halichoeres bivattutus*	slippery dick	Reef	1.82	1.68
*Haemulon aurolineatum*	tomtate	Reef	0.48	0.00
*Monacanthus* sp.	filefish	Reef	0.09	0.00
Pomacentridae	damselfish	Reef	14.47	0.24
*Chromis enchrysurus*	yellowtail reeffish	Reef	0.77	0.00
*Chromis scotti*	purple chromis	Reef	14.52	0.00
*Stegastes fuscus*	dusky damselfish	Reef	9.10	0.00
*Rhomboplites aurorubens*	vermilion snapper	Reef	0.78	10.49
Scorpaenidae	scorpionfishes	Reef	2.38	0.95
Serranidae	groupers	Reef	2.82	0.00
*Baldwinella vivanus*	red barbier	Reef	0.01	0.00
*Centropristis ocyurus*	bank seabass	Reef	7.86	9.41
*Centropristis* sp.	seabass	Reef	0.00	0.14
*Serranus subligarius*	belted sandfish	Reef	0.27	0.00
Total Reef Fishes			89.48	23.96
Non-Reef Benthic Fishes				
*Diplectrum formosum*	sand perch	Non-reef	0.00	6.19
*Diplectrum* sp.	sand perch	Non-reef	0.00	3.60
*Paralichthys albigutta*	gulf flounder	Non-reef	0.00	0.14
Paralichthyidae	large-tooth flounders	Non-reef	0.57	2.78
Pleuronectidae	righteye flounders	Non-reef	0.00	0.05
Pleuronectiformes	flatfishes	Non-reef	0.00	0.02
*Prionotus carolinus*	northern searobin	Non-reef	0.00	0.08
Synodontidae	lizardfishes	Non-reef	1.61	22.22
*Synodus synodus*	diamond lizardfish	Non-reef	0.00	1.28
Triglidae	searobins	Non-reef	0.30	0.44
*Bellator brachychir*	shortfin searobin	Non-reef	0.00	0.27
*Xyrichtys novacula*	pearly razorfish	Non-reef	0.00	5.76
Total Non-Reef Benthic Fishes		2.48	42.81	
Pelagic Fishes				
Carangidae	jacks	Non-reef	1.27	1.59
*Decapterus punctatus*	mackerel scad	Non-reef	0.00	3.43
*Decapterus* sp.	scad	Non-reef	0.00	3.62
*Trachurus lathami*	rough scad	Non-reef	0.00	7.64
Total Pelagic Fishes			1.27	16.28

The overall percent diet by mass is given for natural (NR) and artificial reef (AR) samples.

**Figure 5:**
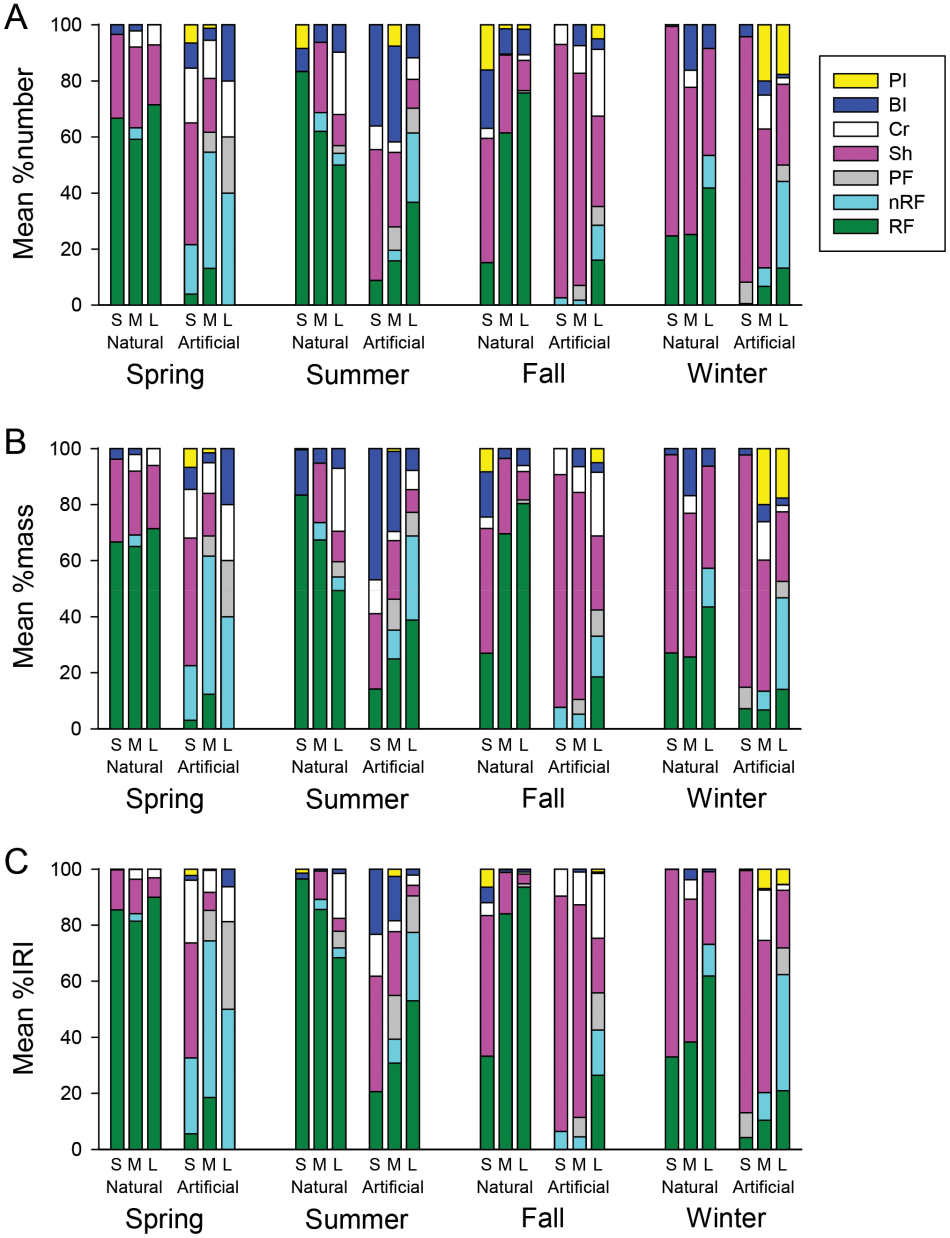
Indices of lionfish diet by habitat, season and size class. Stacked bar plots of A) mean percent diet by number of prey items, B) mean percent diet by prey mass, and C) mean percent index of relative importance for seven prey categories (PI  =  pelagic invertebrates, BI  =  other benthic invertebrates, Cr  =  crabs, Sh  =  shrimps, PF  =  pelagic fishes, nrF  =  non-reef fishes, and RF  =  reef fishes) observed in lionfish stomach samples. Lionfish were sampled with spears in the northern Gulf of Mexico during spring 2013 through winter 14. Size categories: S  =  <200 mm total length (TL), M = 200–250 mm TL, and L = >250 mm TL. Habitat types: natural  =  natural reefs, artificial  =  artificial reefs.

Similar patterns were observed in lionfish diet among prey categories whether %M, %N, or %IRI was considered (Fig. 5). This was due to the fact that there was not a wide range in the sizes of prey items observed. For example, crabs were often similar in size to benthic fishes, and no zooplankton or similar-sized prey were observed in lionfish stomach samples. Habitat type, season, and size class all were significant (p<0.001) in PERMANOVA models that tested for diet differences by %M or %N (Table 2). However, the interactions between habitat type and season (PERMANOVA, p<0.001), as well as between season and size class (PERMANOVA, p = 0.020), were significant in the %M model, and the interactions between habitat type and season (PERMANOVA, p<0.001) and habitat type and size class (PERMANOVA, p = 0.022) were significant for %N.

**Table 2:** PERMANOVA table of factors affecting lionfish diet by prey mass.

Source	df	SS	MS	pseudo-F	p-value
Habitat	1	101,040	101,040	38.14	<0.001
Season	3	66,913	22,304	8.42	<0.001
Size	2	35,809	17,905	6.76	<0.001
H×Se	3	33,589	11,196	4.27	<0.001
H×Sc	2	9,595	4,798	1.81	0.075
Q×Sc	6	27,807	4,635	1.75	0.020
H×Se×Sc	6	23,314	3,886	1.47	0.073
Residual	435	115,220	2,649		

Results from 3-factor permutational analysis of variance model testing the effect of habitat type (natural versus artificial reefs), season, or fish size class (<200, 200–250, or >250 mm total length) on lionfish diet by percent mass. Abbreviations: df  =  degrees of freedom, SS  =  type III sums of squares, MS  =  mean square error, H  =  habitat type, Se  =  Season, Sc  =  size class.

When the habitat type x season interaction in the %M model was sliced by season, there were significant differences in diet between artificial and natural reefs in each season (PERMANOVA, p<0.016). However, when the same interaction was sliced by habitat, there were significant differences in diet for artificial reef samples among all seasons (PERMANOVA, p<0.001) except for between fall and winter (PERMANOVA, p = 0.180). The pattern was different for natural reef samples; diet was only significantly different between winter and the other seasons (PERMANOVA, p<0.011). The same results were observed when the habitat type x season interaction in the %N model was sliced by habitat or season, although the p-values were slightly different than for %M. Overall, these patterns reflect a more constant diet of small demersal reef fishes displayed by lionfish sampled at natural reefs, versus a more varied diet at artificial reefs. The significant difference observed for natural reef samples in winter versus other months reflects a higher percentage of shrimps in that season (Fig. 5).

The season x size class interaction sliced by season for the %M model revealed significant differences among all size classes in fall and winter (PERMANOVA, p<0.036) but no differences in spring or summer (PERMANOVA, p>0.058). When the same interaction was sliced by size class, there were significant differences in diet between all seasons (PERMANOVA, p<0.022) except fall and winter (PERMANOVA, p>0.112) for small and medium sized fish (PERMANOVA, p<0.022). However, the only differences for large fish occurred between winter and spring (PERMANOVA, p = 0.008) and fall and winter (PERMANOVA, p = 0.005). The differences observed for small and medium fish among seasons were mostly due to fluctuations in the percentage of their diets constituted by small demersal reef fishes, while the differences observed for large fish were mostly due to an increase in shrimp consumption in winter.

The habitat x size class interaction sliced by habitat for the %N model revealed significant differences among all size class at artificial reefs (PERMANOVA, p<0.004) but no differences among size classes at natural reefs (PERMANOVA, p>0.083). There were also significant differences between reef types for each of the three size classes (PERMANOVA, p<0.001). Overall, these results reflect the more variable diet observed for lionfish sampled at artificial versus natural reefs.

### Muscle Stable Isotope Analysis

Lionfish sampled for muscle isotope analysis ranged in size between 78 and 363 mm TL (mean TL±95% CI = 224.7±28.9 mm). Total length of these samples was not significantly different between seasons (ANOVA, F_1;28_ = 1.305, p = 0.263) or habitats (ANOVA, F_1;28_ = 0.150, p = 0.701). Correlations between TL and δ^15^N (Pearson's r = 0.79; p<0.001) and TL and δ^13^C (Pearson's r = 0.41; p = 0.006) were both significant. Therefore, the effect of TL on both δ^15^N and δ^13^C was removed by subtracting the slope of the linear relationship between each variable and TL; hereafter, δ^15^N and δ^13^C refer to TL-corrected values.

**Figure 6:**
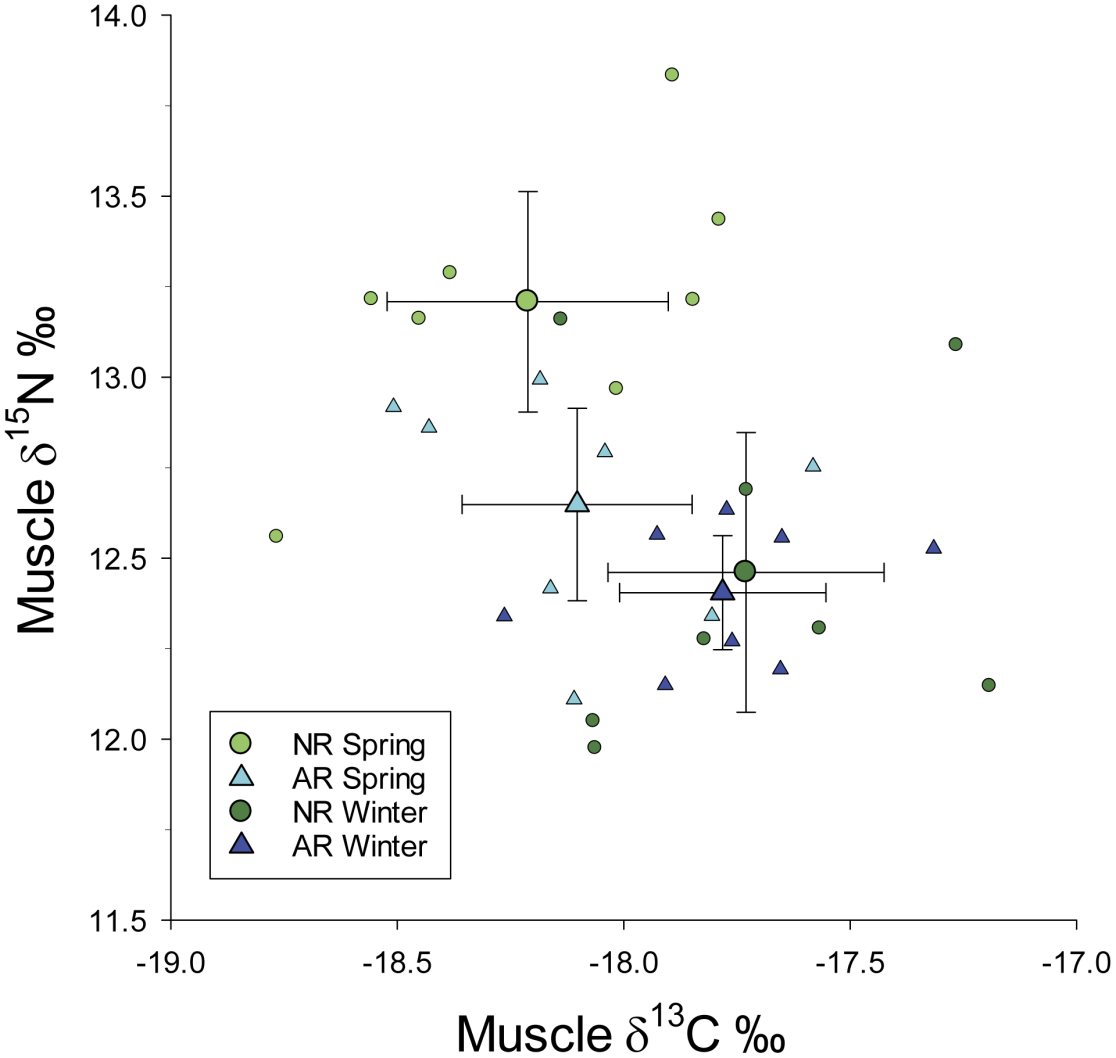
Bi-plot of lionfish muscle δ^15^N versus δ^13^C values. Plot of total length-corrected δ^15^N and δ^13^C values from lionfish white muscle samples collected at northern Gulf of Mexico natural (NR) and artificial (AR) reefs in spring 2013 and winter 2014. Mean values of δ^15^N and δ^13^C are depicted with 95% CI by the four combinations of season and habitat.

Both habitat type (ANOVA, F_1;28_ = 6.29, p = 0.018) and season (ANOVA, F_1;28_ = 16.28, p<0.001) significantly affected lionfish muscle δ^15^N, but their interaction also was significant (ANOVA, F_1;28_ = 4.29, p = 0.050) (Figure 6). Results of Holm-Sidak tests indicated the habitat effect was significant in spring (p = 0.003) but not winter (p = 0.750), and the season effect was significant for natural reefs (p<0.001) but not artificial reefs (p = 0.172). The effect of sampling season was significant for muscle δ^13^C (ANOVA, F_1;28_ = 11.80, p = 0.002), but neither the habitat effect (ANOVA, F_1;28_ = 0.06, p = 0.808) nor the interaction between habitat and season (ANOVA, F_1;28_ = 4.70, p = 0.499) was significant (Figure 6).

## Discussion

The time series of invasive lionfish density estimates reported here indicates an exponential increase in their population size since first being observed in the nGOM in summer 2010. In fact, by fall 2013 mean lionfish density at study artificial reef sites was among the highest reported in the western Atlantic [30], [31], [46]. For example, Hackerott et al. [46], reported lionfish densities between 0 and 52 fish 100 m^−2^ among reefs in Belize, Cuba, and The Bahamas. However, mean density among all their samples (n = 71) was only 4.4 fish 100 m^−2^. Mean lionfish density was several fold higher on nGOM artificial reefs reported here, but even more remarkable is the fact that those densities were reached in only 3 y since lionfish were first observed in this region. Furthermore, the rapid growth of individuals indicates that lionfish biomass, hence prey demand, is increasing even more rapidly in the region than their population growth in numbers.

Factors that have facilitated the expansion of invasive lionfish throughout the western Atlantic are well documented, and include the presence of venomous spines, a voracious appetite, fast growth, early maturity, pelagic egg masses, and historically low abundances of native piscivores in much of the invaded range [25], [47], [23], [27]. It is unclear what mechanisms have facilitated the extremely rapid increase in lionfish densities in the nGOM or why lionfish densities on artificial reefs are two orders of magnitude higher than on natural reefs, although high lionfish densities have been reported on manmade structures in other systems [48], [49]. The highest lionfish densities reported among Caribbean reefs surveyed by Hackerott et al. [46] occurred at patch reefs in The Bahamas, and artificial reefs in the nGOM likely mimic attributes of patch reefs versus more expansive types of natural reefs. Artificial reefs deployed in the nGOM tend to be isolated concrete modules, as were the bulk of reefs examined in the current study, or other sunken manmade materials, like shipwrecks or surplussed military vehicles [38], [39]. Similar to coralline patch reefs [46], nGOM artificial reefs also tend to have a footprint on the scale of 10^2^–10^3^ m^2^ and vertical relief that is substantially higher (typically 2–3 m) than the surrounding seabed [39]. Multiple large (>200 km^2^) artificial reef permit areas on the shallow (<50 m) nGOM shelf facilitate widespread artificial reef deployment in the region, but reef modules or other types of manmade structure tend to occur on sandy or muddy substrates with adjacent artificial reefs often being >500 m apart [50], [51], [38]. In areas of the shelf lacking natural reef structure, settling larval fishes cue to high vertical relief [48], [52], thus the patchy distribution of artificial reefs may serve to concentrate settling juvenile lionfish.

Predator-prey dynamics also may affect the distribution of lionfish on the nGOM shelf, although perhaps in unexpected ways. Circumstantial and direct evidence exists that large (>10 kg) piscivores, such as sharks or groupers, may consume adult lionfish in some parts of their invaded range [53], [54], [55]. However, Patterson et al. [39] reported that large piscivores actually had higher densities on artificial versus natural reefs in the nGOM. Artificial reef communities in the region are dominated (up to 25% by number and 40% by biomass) by red snapper, *Lutjanus campechanus* [38], but no lionfish have been observed in red snapper stomach samples [56]. Groupers (family: Serranidae) tend to be twice as abundant on natural versus artificial reefs in the system, but their density is an order of magnitude lower than that of snappers (family: Lutjanidae) [39]. Perhaps lionfish population control by native piscivores would be more likely to occur via predation on early life stages versus adult lionfish [57], [58], but no direct observation of that has been reported to date.

Throughout their invaded range, diet analyses have demonstrated invasive lionfish to be generalist mesopredators with a preference for small (<5 cm) demersal reef fish prey [34], [36]. Among our samples, crustaceans and other invertebrates were more important contributors to the diet of smaller (<200 mm) lionfish, which has been reported from other regions [16], [49]. Piscivory clearly increased as lionfish grew, although the contribution of fish to lionfish diet was lower on artificial versus natural reefs. The ontogenetic shift to greater piscivory observed among diet samples was corroborated via δ^15^N analysis of muscle tissue, given the positive correlation between δ^15^N and TL and the fact that δ^15^N increases with trophic position due to trophic fractionation [59]. Furthermore, the highest muscle δ^15^N values occurred among the largest (>250 mm TL) lionfish sampled at natural reefs in spring 2013 which corresponded to the highest degree of piscivory observed among diet samples.

Small demersal fishes, such as damselfishes (family: Pomacentridae), blennies (family: Blenniidae), gobies (family: Gobiidae), and wrasses (family: Labridae) are among the more numerically dominant taxa on nGOM natural reefs but are nearly absent from artificial reef communities [39]. Therefore, higher abundances of small demersal reef fishes on natural versus artificial reefs also would seem to favor higher densities of lionfish on natural reefs, but the opposite pattern was observed. Lionfish sampled at nGOM artificial reefs tended to have more varied diets than on natural reefs, with fish foraging on a higher proportion of non-reef and invertebrate prey. Therefore, the lack of small demersal reef fish prey on artificial reefs did not seem to be a limiting factor with respect to lionfish density.

Clearly, the generalist nature of lionfish foraging can extend well beyond simply feeding on a variety of small reef fishes. That was especially true of lionfish sampled at artificial reefs in fall and winter when invertebrate prey constituted >50% of their diet, even for the largest (>250 mm TL) fish. Those trends were supported by muscle δ^15^N values that were lower in winter when fish were feeding on lower trophic level prey, and also lower for lionfish samples collected at artificial versus natural reefs. Values of muscle δ^13^C were not significantly different between reef types, but there was a significant season effect in which δ^13^C was lower in winter than spring samples. Again, this corroborates diet data in that benthic invertebrates are likely to have lower δ^13^C values due to benthic microalgae being depleted in ^13^C relative to phytoplankton [60].

Invasive lionfish have the potential to cause substantial ecosystem impacts in the nGOM given their density, feeding ecology, and growth rates [27], [35], [61]. However, another concern for resource managers is their potential impact on exploited species. Few exploited fishes were observed within lionfish stomach samples, but among them were flounders (families: Paralichthyidae and Plueronectidae) and vermilion snapper, *Rhomboplites aurorubens*. While few reef fish taxa were observed in lionfish stomachs at artificial reef sites, vermilion snapper was present in summer, fall, and winter samples and constituted 10.5% of lionfish diet by mass at artificial reefs. Many of the exploited species common to both natural and artificial reefs in the nGOM initially settle out of the plankton in other habitats and then recruit to reefs later in life [62]. Fishes that settle out of the plankton directly onto reefs, such as vermilion snapper, likely will be much more vulnerable to direct lionfish impacts than species that recruit to reefs as older individuals.

Being ecological generalists, in terms of habitat and/or dietary preferences, is a characteristic shared among the most successful of fish invaders [63]. Although lionfish had a broad diet among all habitats, those sampled from artificial reefs fed on a wider variety of prey resources, the majority of which were non-reef associated prey inhabiting nearby sandy substrates. This pattern likely stems from higher lionfish densities on artificial reefs that may have depleted available reef prey [34], or increased intra-specific competition [64] forcing individuals away from reefs to forage. The likelihood of food limitation for lionfish would be inherently greater at artificial reefs if their diet was restricted to small demersal reef fishes, given that these fishes are less abundant and diverse on local artificial reefs than on natural reefs [38], [39]. Ultimately, the high densities of lionfish at artificial reef sites, coupled with abundant non-reef associated taxa in their stomachs, demonstrate their ability to forage on open substrates away from reefs. Movement of lionfish with respect to foraging behavior may vary widely depending on the characteristics of a given site. For example, lionfish have been observed traveling away from coral patch reefs during foraging bouts in the Bahamas [17], yet in an estuarine system, researchers found that lionfish display high site fidelity [65]. Consistent with earlier investigations of lionfish trophic ecology, our results suggest lionfish are ecological generalists in the nGOM and illustrate their adaptability to a range of habitat [49], [66] and foraging conditions [16], [36].

Northern GOM lionfish populations likely have not yet reached their peak, as estimates of density, TL, and body mass increased throughout the study period, and lionfish density on artificial reefs is two orders of magnitude higher than on natural reefs. Juvenile lionfish have been shown to exhibit density-dependent growth on artificial patch reefs in The Bahamas [67], but it is unknown whether lionfish densities are sufficiently high relative to food resources to cause a similar negative feedback in the nGOM. Sample sizes from ROV sampling were insufficient to test for differences in lionfish TL between natural and artificial reefs, but there was a significant difference in fish size among years. Total length distributions of lionfish sampled by spear also shifted to larger sizes from spring 2013 to winter 2014, which is consistent with larger fish becoming more predominant in the system. The presence of multiple TL modes among lionfish sampled with spears also indicates the presence of multiple year classes in each of the sampled habitats and, the increase in the number of individuals <150 mm TL in winter 2014 may indicate local self-recruitment is occurring. Self-recruitment would imply that invasive lionfish populations now clearly established in the nGOM would not require recruitment from other regions to ensure population persistence or growth. Furthermore, entrainment of eggs and larvae in the Gulf Loop Current from nGOM spawning events may implicate this region as a source of recruits to downstream regions such as the west Florida shelf or the Florida Keys [68], [69].

The potential for negative ecological impacts is likely to increase as lionfish populations expand in the nGOM. Although predation on adult lionfish by large piscivores has been inferred or observed in some regions [53], [54], [55], native predator density has not impacted lionfish colonization or population density in the Caribbean region [46]. Lionfish have enormous potential to negatively affect native communities either by consuming fauna directly or competing with native predators for the same forage base or space on reefs. Native groupers and snappers have habitat preferences similar to those of lionfish, thus examining reef fish behavior and movement on reefs with respect to lionfish presence could reveal indirect effects on these native fauna. The infrequency with which empty lionfish stomachs were encountered in this study implies highly successful feeding [37], and may indicate the naivety of native prey species to lionfish presence. Reductions in the abundance of reef and non-reef associated small demersal fishes due to lionfish predation may have far reaching impacts on nGOM reef ecosystems. In such cases that prey resources become depleted, it would be useful for researchers to monitor changes in lionfish foraging behavior and site fidelity, both of which have the potential to impact the effectiveness of lionfish mitigation efforts. In addition, future research should also be focused on tracking changes in lionfish density over time and examining their bioenergetic demands, direct consumption of native reef fishes, and growth rates.
